# Silent Chiari Type I Malformation Presenting With Markedly Asymmetrical Papilledema Detected on Routine Ophthalmic Examination: A Pediatric Case Report

**DOI:** 10.7759/cureus.101761

**Published:** 2026-01-18

**Authors:** Doğukan Cömerter, Feyza Rumeysa Öz, Dilara Ayaz Kaya

**Affiliations:** 1 Department of Ophthalmology, Sultan Abdulhamid Han Training and Research Hospital, University of Health Sciences, Istanbul, TUR

**Keywords:** asymmetrical papilledema, chiari malformation type 1, hydrocephalus, optic disc edema, pediatric neuro-ophthalmology

## Abstract

Chiari type I malformation (CMI) is a congenital hindbrain anomaly that may disrupt cerebrospinal fluid (CSF) dynamics and lead to intracranial hypertension. Although papilledema is a well-recognized manifestation of elevated intracranial pressure, it is typically bilateral and symmetric. Asymmetrical papilledema is uncommon, particularly in pediatric patients, and may pose a diagnostic challenge.

We report a 10-year-old boy with no neurological or visual complaints who was referred after asymmetrical optic disc edema was detected during a routine ophthalmic examination. Visual acuity was preserved bilaterally; however, fundus examination revealed mild papilledema in the right eye and pronounced papilledema in the left eye. Optical coherence tomography demonstrated markedly asymmetric thickening of the peripapillary retinal nerve fiber layer. Neuroimaging revealed a CMI associated with severe triventricular hydrocephalus. The patient underwent endoscopic third ventriculostomy, resulting in gradual resolution of papilledema. Despite preserved visual acuity, secondary optic atrophy developed in the initially more severely affected eye, defined clinically by optic disc pallor and supported by retinal nerve fiber layer thinning on optical coherence tomography.

This case highlights that silent CMI with hydrocephalus may present solely with markedly asymmetrical papilledema in an otherwise asymptomatic child. Routine ophthalmic examination can play a pivotal role in the early detection of clinically occult but potentially vision-threatening intracranial pathology.

## Introduction

Chiari type I malformation (CMI) is a congenital abnormality characterized by downward displacement of the cerebellar tonsils below the foramen magnum. This anatomical alteration may interfere with cerebrospinal fluid circulation at the craniocervical junction, leading to a wide range of neurological manifestations. While symptoms such as occipital headache, cranial nerve dysfunction, and cerebellar signs are frequently described, pediatric patients may remain asymptomatic or present with nonspecific findings for extended periods [1].

In some cases, increased intracranial pressure related to CMI may be reflected only by ophthalmic findings. Papilledema represents optic disc swelling secondary to elevated intracranial pressure and is typically bilateral. However, asymmetrical papilledema has been reported, particularly in children, and may delay recognition of the underlying pathology [2]. In such situations, routine ophthalmic examination may be the first indication of an intracranial disorder.

Here, we report a pediatric case in which markedly asymmetrical papilledema detected during routine ophthalmic evaluation led to the diagnosis of silent CMI with associated hydrocephalus. Markedly asymmetrical papilledema is rarely reported in pediatric patients and represents a diagnostic challenge, particularly in the absence of neurological symptoms. This case highlights a clinically silent CMI presenting solely with pronounced inter-eye asymmetry of papilledema.

## Case presentation

A 10-year-old boy with no history of systemic or neurological disease presented for a routine ophthalmic examination. He denied visual complaints, headaches, or neurological symptoms. Best-corrected visual acuity was 20/20 in both eyes. Ocular motility was full and orthophoric, and anterior segment examination was unremarkable. Color vision was assessed using a red desaturation test, which demonstrated a relative reduction in the left eye. Formal automated visual field testing could not be reliably performed due to the patient’s young age and limited cooperation.

**Figure 1:**
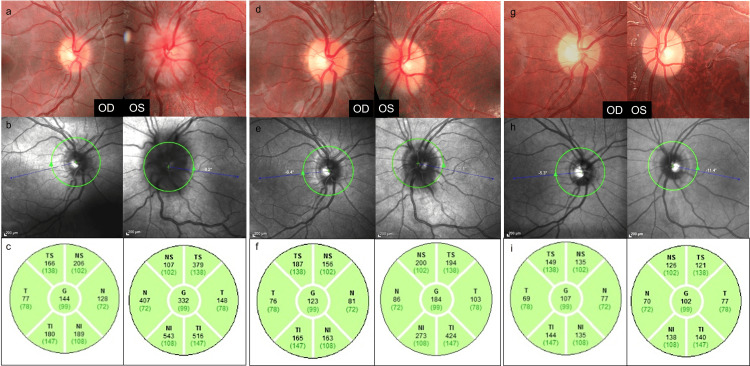
Multimodal ophthalmic imaging demonstrating the evolution of markedly asymmetrical papilledema in a pediatric patient with Chiari type I malformation and hydrocephalus. Color fundus photographs (a, d, g), near-infrared reflectance images (b, e, h), and peripapillary retinal nerve fiber layer thickness maps obtained by optical coherence tomography (c, f, i) are shown at presentation, one month after endoscopic third ventriculostomy, and at the 8-month follow-up. At presentation, fundus photography shows mild optic disc edema in the right eye and pronounced papilledema in the left eye, with corresponding asymmetric retinal nerve fiber layer thickening. Following endoscopic third ventriculostomy, gradual bilateral resolution of papilledema is observed, with normalization of retinal nerve fiber layer thickness over time.

Dilated fundus examination revealed marked asymmetry between the optic discs. The right optic disc showed mild margin blurring with minimal elevation, whereas the left optic disc demonstrated pronounced swelling. According to the Frisén scale, papilledema was graded as stage 1 in the right eye and stage 3 in the left eye. Optical coherence tomography revealed peripapillary retinal nerve fiber layer thicknesses of 144 μm in the right eye and 332 μm in the left eye. Fundus autofluorescence imaging showed no evidence of optic disc drusen (Figure 1).

**Figure 2:**
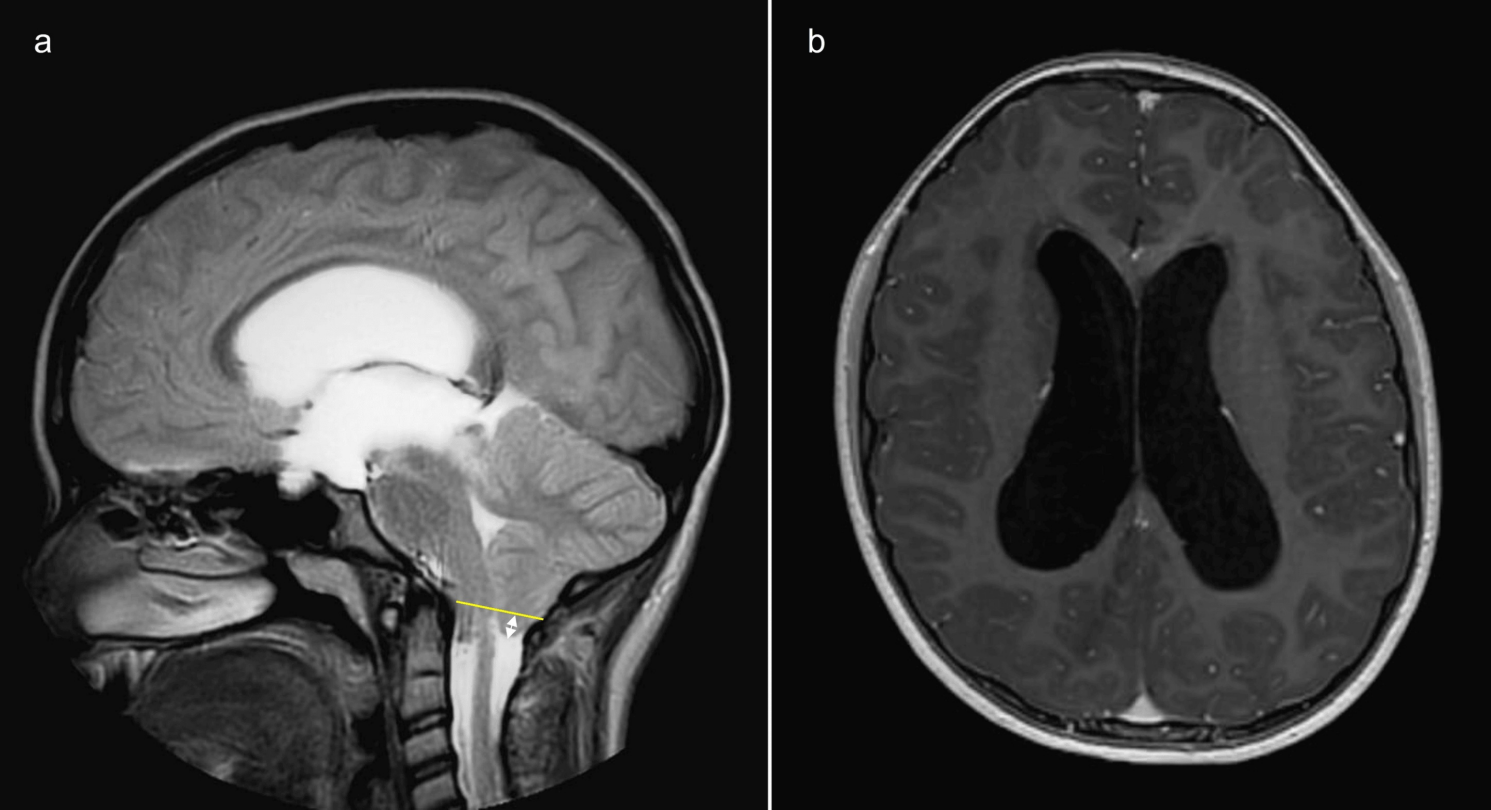
MRI findings in a pediatric patient with Chiari type I malformation and hydrocephalus. (a) Sagittal T2-weighted MRI demonstrates caudal herniation of the cerebellar tonsils below the level of the foramen magnum (arrow), consistent with Chiari type I malformation, with associated crowding at the craniocervical junction. (b) Axial T1-weighted MRI shows marked dilatation of the lateral ventricles, consistent with triventricular hydrocephalus.

To distinguish papilledema from pseudopapilledema, cranial and orbital magnetic resonance imaging was performed. Neuroimaging revealed a CMI with caudal herniation of the cerebellar tonsils and severe triventricular hydrocephalus, consistent with obstructive (non-communicating) hydrocephalus due to impaired cerebrospinal fluid flow at the craniocervical junction. Crowding at the foramen magnum was present, with no evidence of aqueductal stenosis (Figure 2).

The patient was referred to pediatric neurology and neurosurgery, and endoscopic third ventriculostomy was recommended. Endoscopic third ventriculostomy was selected to restore physiological cerebrospinal fluid circulation and to avoid long-term shunt dependency in a pediatric patient with obstructive hydrocephalus.

Endoscopic third ventriculostomy was performed approximately four months after the initial ophthalmic assessment. At one-month follow-up, visual acuity remained preserved in both eyes. Fundus examination showed complete resolution of papilledema in the right eye and marked improvement in the left eye. Retinal nerve fiber layer thickness decreased to 123 μm in the right eye and 184 μm in the left eye. At four months postoperatively, optic disc appearance was clinically normal in both eyes, with retinal nerve fiber layer measurements of 107 μm and 102 μm, respectively. Postoperative retinal nerve fiber layer thickness values were within reported normative pediatric ranges, supporting resolution of optic disc edema (Figure 1).

At the tenth postoperative month, visual acuity remained stable in both eyes. Supportive neuroprotective therapy with citicoline-containing micronutritional supplementation was initiated. During subsequent follow-up, visual acuity and retinal nerve fiber layer thickness remained stable.

## Discussion

Papilledema results from elevated intracranial pressure transmitted to the optic nerve head and is typically symmetric. Asymmetrical presentations, although uncommon, have been described in pediatric patients and may relate to anatomical variability of the optic nerve sheath, differences in subarachnoid space compliance, or uneven cerebrospinal fluid pressure transmission [3]. The rarity of such cases in pediatric series highlights the clinical relevance of our report.

This case demonstrates an unusual presentation in which a completely asymptomatic child was diagnosed with CMI and hydrocephalus solely based on markedly asymmetrical papilledema detected during a routine ophthalmic examination. The absence of neurological symptoms and the subtle involvement of the contralateral eye emphasize how such cases may be overlooked without careful fundus evaluation. Routine ophthalmic examinations may occasionally represent the first opportunity to detect clinically silent intracranial pathology in pediatric patients.

Hydrocephalus occurs more frequently in children with CMI than in adults and plays a central role in the development of intracranial hypertension. Impaired cerebrospinal fluid flow at the craniocervical junction may result in ventricular dilatation and sustained pressure elevation [4]. Endoscopic third ventriculostomy provides a physiologic alternative to shunt placement by restoring cerebrospinal fluid circulation and reducing long-term shunt dependency. In the present case, this approach was associated with resolution of papilledema and preservation of visual function.

Asymmetrical papilledema may represent a diagnostic pitfall in pediatric patients, particularly when visual acuity and neurological examination are normal. Optical coherence tomography may be less sensitive in detecting very early or subtle papilledema; however, careful assessment of inter-eye asymmetry, supported by optical coherence tomography, can facilitate early recognition of increased intracranial pressure and guide timely neuroimaging.

Secondary optic atrophy was defined clinically by optic disc pallor and supported by thinning of the retinal nerve fiber layer on optical coherence tomography. The development of secondary optic atrophy in the initially more severely affected eye highlights that the duration and severity of papilledema may lead to irreversible optic nerve damage, even when visual acuity is preserved [5]. This finding emphasizes the importance of early recognition, prompt neuroimaging, and close multidisciplinary follow-up in pediatric patients with unexplained papilledema.

To our knowledge, presentation of a clinically silent CMI with severe hydrocephalus solely through markedly asymmetrical papilledema in an otherwise asymptomatic child is exceptionally rare.

## Conclusions

This case demonstrates that CMI with associated hydrocephalus may remain clinically silent in pediatric patients and present exclusively with markedly asymmetrical papilledema. Routine ophthalmic examination can be crucial in identifying occult intracranial pathology before the onset of neurological symptoms. Early diagnosis and timely neurosurgical intervention are essential to prevent permanent optic nerve damage and preserve long-term visual function.
